# “Too big to fail”: the resilience and entrenchment of food aid through food banks in the Netherlands during the COVID-19 pandemic

**DOI:** 10.1007/s12571-022-01260-5

**Published:** 2022-02-03

**Authors:** Paulien Dekkinga, Hilje van der Horst, Thirza Andriessen

**Affiliations:** Consumption and Healthy Lifestyles, Wageningen University & Research, P.O. Box 8130, 6700EW Wageningen, Netherlands

**Keywords:** Food banks, Resilience, COVID-19, Food aid, Food systems, The Netherlands

## Abstract

This paper aims to better understand the resilience and further entrenchment of food aid through food banks in the context of the COVID-19 pandemic. During the first months of the pandemic in the Netherlands, concerns quickly rose about the number of people falling into conditions of food insecurity. Adding insult to injury, food banks reported problems in their operations. The analysis shows that after some adaptations to initial problems, food banks were largely able to continue their service. This ability was partly based on organizational flexibility. However, in order to understand the resilience of food aid through food banks, it is imperative to understand food banks as part of a system of food aid that extends beyond the organizational boundaries. This system includes a range of other actors and resources, including donors, public support and governmental backing that contributed to the resilience of the food aid system. While this embeddedness in a system as well as broad public support were essential for the resilience of food aid through food banks, both factors also indicate the further entrenchment of food banks in the understanding and practices of ensuring food security for people in poverty. Ultimately, when the root causes of a need for food aid are not addressed, a resilient system of food aid through food banks can eventually prove detrimental to societal resilience, specifically the ability to ensure dignified access to adequate food.

## Introduction

This article sets out to unravel and explain the resilience of food aid through food banks under the challenging conditions during the first months of the COVID-19 pandemic. There have been urgent concerns about the adequacy and reliability of a charitable, volunteer-based organization to maintain operations in the current context with unparalleled challenges felt throughout societies (Candel & De Zwarte, 2020). As stated by Fleetwood (2020), these challenges test our societal commitment to ensure the human right to sufficient, safe and nutritious food, as part of the Sustainable Development Goals (SDGs).

Essential practices on which food banks depend to provide emergency food were first challenged by the impact of the pandemic and related measures on a range of essential practices (Baker and Russell, 2020; Penco et al., 2021). Such practices include food transport and fundraising events as well as food transfer and food salvaging. As indicated by Power et al. (2020), practices to provide emergency food are also jeopardized because of disrupted food chains in the broader food system. A second challenge posed to food banks and other food aid organizations was the increased demand for food aid. The COVID-19 pandemic and associated measures have had substantial economic impacts (Vieira et al., 2020; WHO, 2020). This has also been the case for the Netherlands, where many shops, hospitality and catering industry had to close (Antonides & van Leeuwen, 2020). Although the Dutch government invested in social benefit allowances and other measures to maintain jobs, such as providing compensations if companies remained all personnel on the job (Antonides & van Leeuwen, 2020), still unemployment went up from 3.8% in the second quarter of 2020 to 4.5% in the third quarter of 2020 (CBS, 2020). As a consequence of such economic impacts, more households became dependent on food aid to put enough food on the table (The Lancet, 2020; Loopstra, 2020; Wolfson & Leung, 2020). In December 2020, an increase of 7.2% in the number of recipients of Dutch food banks was noticed compared to 2019 (Voedselbanken Nederland, 2021).

Those two challenges not only raise the question of how resilient food assistance through food banks was during the first months of the COVID-19 pandemic, but also what factors contributed to resilience or the lack thereof. As we will argue, a study of resilience needs to focus on both the organization itself and the broader embeddedness within a societal system of food assistance (Galli et al., 2018). This system includes the organizations that distribute food to households, but also the role played by governments and other outside actors. In line with two case studies in Italy and Britain about food banks during the corona pandemic (Baker & Russell, 2020; Penco et al., 2021), our research shows that food aid through food banks was challenged by various disruptions during the first months of the pandemic. However, food banks as embedded in the broader system they are part of proved its adaptive capacity. It also shows that the resilience of this system of food aid can only be explained by food banks having emerged as a dominant system for food assistance that numerous actors, including governments, support. However, while food aid through food banks appeared rather resilient, this cannot be mistaken as a resilient society that ensures safe, sufficient and dignified access to food to all inhabitants.

Over the last decades, charitable food aid through food banks has become a dominant system of food aid in European countries (Lambie-Mumford & Silvasti, 2020) and are increasingly relied on as a structural addition to arrangements of the welfare state, especially in cases where such arrangements fail to prevent food insecurity (Kessl et al., 2020). Within debates about the position of this charitable system, governments are criticized for allowing situations of food insecurity to exist and not mending the gaps in the welfare system. Those critiques range from the impact on the dignity of food aid recipients (van der Horst et al., 2014; Garthwaite, 2016; Power, 2011; Riches & Silvasti, 2014; Andriessen et al., 2020) to their inadequacy to provide access to safe and nutritious foods to populations in need (Neter et al., 2016; Depa et al., 2018). Overarching these critiques is the notion of a government retreating from its obligations to ensure a right to food by leaving the responsibility to provide access to food to people in poverty to a third sector organization (Riches & Silvasti, 2014).

The next section provides a theoretical discussion of systems of food aid and resilience. We then provide an overview of the methods of the research. Following the theoretical discussion, we structure the results according to the four elements of systems of food aid, namely actors, resources, processes and functions. In the conclusion, we discuss the implications of this research for understanding the entrenchment of food banks in societal responses to food insecurity and the implications for the resilience of a society.

## Systems of food assistance and resilience

We aim to gain a better understanding of the resilience of food aid through food banks. In order to achieve this understanding, we propose that it is necessary to understand food banks as part of a system of food aid. A system of food aid includes organizations offering food, but also includes the consumers donating food during food drives at a supermarket, local churches giving monetary donations, governments allowing the use of facilities and a food system that generates surpluses that can be repurposed as food aid. Galli et al. (2018) offer a helpful overview of the diverse elements that are involved in a system of food assistance based on three case studies of food assistance. According to the authors, food assistance systems consist of four elements: (i) actors, (ii) resources, (iii) functions, and (iv) processes. We use this helpful distinction to better understand whether and how the system of food aid based on food banks showed resilience.

To start with *actors*, the relevant actors include volunteers, donors, ambassadors, local government, national government, and charity-oriented organisations (Bacon & Barker, 2017; Galli et al., 2018). The second element consists of the *resources* food banks use to provide food assistance, which includes food surplus, food donations, monetary donations, and time spent by volunteers (Galli et al., 2018; González-Torre & Coque, 2016). The *functions* that food banks fulfil form the third element. Besides the evident function of providing emergency food aid, food banks often provide a place for social interaction with an informal, approachable ambiance where recipients can interact with others in similar situations of poverty and food insecurity (Rosenthal & Newman, 2019; Salonen, 2017). Additionally, food banks can fulfil a function of reducing food waste (Galli et al., 2018; van der Horst et al., 2014). The last element comprises the *processes* that take place in the food assistance system. Such processes are for example food (re-)distribution, food recovery, and guarding of food safety (Galli et al., 2018).

Having established what a system of food aid entails, we now turn to resilience. Following the discussion above, we argue that in order to understand the resilience of food aid through food banks, we need to focus not only on distinct food banks and their organizational resilience (Barasa et al., 2018) but also on the broader system of food aid that they are part of.

To this end, we incorporate an understanding of system resilience in our conceptualisation. Food system resilience can be understood as the ability of a food system to deal with unforeseen disruptions while continuing its processes (Gillespie, 2020; Tendall et al., 2015). Tendall et al. (2015) describe three capacities by which a system can react to a shock: absorptive, adaptive, and transformative. The absorptive capacity is the ability to continue as usual in the case of a shock. The adaptive capacity is the ability to adjust and adapt to situations. The transformative capacity is the ability to transform and create a fundamentally new system when necessary. Additionally, preventive actions can be taken to prevent disruptions from future shocks (Tendall et al., 2015).

By combining a system approach to food aid with this discussion of food system resilience, we can define resilience of a system of food aid as the ability of such a system to absorb shocks, to adapt to new circumstances or to transform into a new system, while continuing food assistance. Distinguishing the various elements of systems of food aid, helps top unravel how distinct actors, resources and processes, inside as well as outside the organizations, aid or hinder the continuation of the various functions, with a primary focus on the ability to maintain food assistance.

Earlier research on food aid at a time of crisis underlines the relevance of looking at the embeddedness of food aid organizations in a broader system. In the context of the economic crisis that started in 2007–2008, Coque and González-Torre (2017) provided insight in the adaptive capacity of food banks in Spain. Their research indicated that food banks in Spain were efficiently organized through “*very participative human resources and a solid network of external relationships*” (2017:643). Especially the connections with external organizations and the widespread appreciation for food banks, filling various resource gaps, were marked as contributing to the adaptive capacity of food banks (Coque & González-Torre, 2017). To what extend these results align with capacities of a food aid system in another country, in our case the Netherlands, and in relation to a fundamentally different crisis, as embodied in the corona pandemic, is a topic for further enquiry.

Societal support for food aid is a fundamental factor in the resilience of a system of food aid. Elcheroth and Drury emphasize that in the context of the pandemic, social solidarity and social contact are of vital importance for achieving collective capacities for resilience (2020). Due to lockdown orders, especially the ability to sustain social contact is jeopardized. At the same time, people feel an urge to show solidarity with those who are more vulnerable or who are hit harder by the consequences of the COVID-19 pandemic. While the authors see the pandemic as a time during which solidarity can be strengthened which could contribute to resilience and an ability to emerge stronger from a crisis, they also warn that such resilience may be jeopardised when social interaction lessens and social inequalities are allowed to grow bigger during the pandemic. This warning is especially relevant to our aim of better understanding both the resilience of food aid through food banks and the further entrenchment of a system of food aid through food banks during the pandemic. While food banks can offer much needed and valuable relief to households at a time of crisis, societal resilience ultimately depends on the ability to mend the structural issues that lead to the kinds of inequalities where people become dependent on food aid.

## Methodology

Before turning to the methods we used to better understand the resilience and entrenchment of food banks playing a central role in a system of food aid, some clarity about the Dutch context is necessary. In the Netherlands the term food bank is used both for the places where recipients come to get food (or food pantry), and for the local organizations that may have one or more of such food pantries. Volunteers, donors, recipients, as well as local and national governments are included as actors in the system, because food banks inherently rely on these actors for their resources (Bacon & Barker, 2017; Galli et al., 2018; Voedselbanken Nederland, 2020a).

In addition, it is important to understand the different geographical scales of food bank organizations, which may include a range from local to national level (Galli et al., 2018; Voedselbanken Nederland, 2015). In the Netherlands, local food banks cooperate in a national federation of food banks and regional distribution centres distribute food to food banks in their region, while food banks also collect food locally.

Based on the particular organizational structure of food banks in the Netherlands, in the research we focussed on three scales: (i) the national scale at which the Association of Dutch Food banks coordinates, (ii) the regional scale with the regional distribution centres, and (iii) the local level where the local food banks hand out food to recipients. Food banks in the Netherlands are entirely volunteer based. Semi-structured interviews were conducted with volunteers working at each of those three scales. The interviews were held by phone or video-calls to adhere to the COVID-19 safety measures.

Voedselbanken Nederland published a list of food banks that closed or changed distribution during COVID-19, which gave an explorative overview of the situation food banks were in (Voedselbanken Nederland, 2020b). With the use of this information, food banks with varying responses to the COVID-19 situation were approached, including full closure, a switch to food vouchers, and food delivery. In total, 18 interviews were performed, of which one at national level with a volunteer working for the association of Voedselbanken Nederland, six with volunteers from regional distribution centres, and eleven with volunteers acting at local food banks.

The topic list for the interviews was based on the four elements of food assistance determined by Galli et al. (2018): (i) actors, (ii) resources, (iii) functions, and (iv) processes. All interviews were performed between 29 May 2020 and 30 June 2020. Interviews looked at the period since March 2020, when measures to curb the spread of the virus were first implemented. During the period of the interviews several of the more severe measures had been lifted. After being fully transcribed, the interviews were thematically coded, using the four elements of food assistance by Galli et al. (2018) as the initial coding scheme. Further analysis consisted of identifying how elements and changes therein contributed to the continuation of food provisioning, and the kind of resilience observed.

The results where shared during two food bank meetings in 2021: 1) on March 17, during a meeting with board members at the national level, 2) on May 11, during a webinar open for all volunteers of the 170 local food banks whereby eventually 26 volunteers joined. During these meetings volunteers recognized and confirmed the results, and some volunteers from local food banks additionally shared some specific challenges and adaptations at their local food bank.

## Results

### Actors—loss and gain of volunteers and donors

Actors are the first element in food aid systems. They include those who are directly involved in the food bank organization as well as outside actors. A prominent concern regarding the resilience of food banks is their reliance on volunteers, who are often retired (Candel & De Zwarte, 2020). Indeed, our research showed that COVID-19 had a tremendous impact on the input of volunteers. An interviewee from the national organization estimated that 80% of the volunteers at food banks are older than 60 years. This means a substantial proportion of the volunteers belong to a high-risk category for COVID-19, of whom many stayed home to minimise the risk of getting COVID-19. This resulted in temporary closure of local food banks, but also the regional distribution centres. Especially the latter challenged the continuation of food aid.

*“That first week when there was a lockdown, there was a moment of panic. Of the 170 food banks, about 20 shut down. Additionally, we had three DCs [distribution centres] that were about to collapse. That was Amsterdam, Haaglanden and Tilburg. (…) Those three DCs had stopped, which meant we were suddenly without much stuff, because they deliver to about 50% of our clients.” *(Voedselbanken Nederland)

While the immediate and substantial loss of active volunteers raised concerns about the continuity of food assistance, in fact interviewees reported a substantial interest from younger people to become a volunteer, reflecting findings by Tierney and Mahtani (2020). This increased interest could be explained by a combination of food banks publicising their need for volunteers, with young people being forced to stay home from work and education and seeking a meaningful way to spend their days. As a consequence, multiple food banks got more applications than needed. An interviewee from a local food bank shared experiences with the increase in applications of new volunteers:

*“... We also received all kinds of phone calls from people who said ‘I have nothing to do, I can help’. Or ‘I want to become a volunteer for a longer period of time’. So many people were interested that we said: we only ask the people who want to stay for a longer period of time. So that has actually been a very special phenomenon.”* (Local food bank 5)

The reliance on volunteers has certainly posed challenges to the continuation of food banks, as the first quote shows. But as adding new volunteers to the organization could be done quickly, and due to the public support for food banks, operations were able to absorb the shock of losing older volunteers.

Next to the influx of new volunteers, interviewees noticed an increase as well as a shift in the involvement of donors. The range of donors is diverse and includes private individuals, organization-ambassadors, local governments, and charity-oriented organizations. Especially at the start of the COVID-19 outbreak, new donors offered support in the form of food, money, and helping hands. For instance, the volunteer coordinator and spokesperson of one of the local food banks said about this increased involvement:

*“But that number of sponsors has increased enormously. I can't say percentages, but for the first six weeks [of the COVID-19 pandemic] my colleagues and I worked just about full time because it was so busy with people who wanted to give us money and companies who wanted to help us. So, interest and donations have increased a lot. What has also increased a lot is the donations from individuals. Previously, it was only sometimes that people donated ten or a hundred euros. But now it has been many more individuals in the city who have donated money.”* (Local food bank 4)

Another change in actors was seen in the involvement of government. Many local food banks already have contacts with the municipality and receive various forms of support. Interviewees stressed that at the onset of the pandemic local governments were keen to be informed about the situation of the foodbank in their municipality and offered to support where needed.

*“Well, of course there has been intensive contact with the municipality. Like 'how are you doing?' And we have also committed that if there is anything, we will let the municipality know. So it has also been decided, that if it is really necessary that they help us financially to keep things going.”* (Local food bank 8)

Before COVID-19, the Dutch national government was not directly involved in supporting food banks. However, during the COVID-19 outbreak, the national government set up a guarantee fund of € 4 million that could be used in case of insufficient food supply or increased costs due to the consequences of the pandemic. In addition, defence staff was deployed at three regional distribution centres. They filled in for volunteers who had to stay home in order to ensure the continuation of food assistance and helped with making the halls ‘corona-proof’. While the gesture was appreciated, an interviewee also downplays the numerical significance of military personal.

*“When you see the press releases from the Ministry of Defence, you get the idea that the Ministry of Defence has saved food banks in the Netherlands. We have 12,000 volunteers and we had 12 Defence personnel. Just keep an eye on the ratio. But they did make a difference at those three locations, at that time.” *(Voedselbanken Nederland)

Thirdly, changes were seen and expected in the number of recipients. Although recipients were not explicitly mentioned as an actor in the food assistance system as described by Galli et al. (2018), this research has indicated that the number of recipients in the food assistance system is crucial for the resilience of the food bank system, because it affects to what extend needs can be addressed. An increase in the number of recipients varied between locations, with some food banks experiencing no increase, while others experienced an increase of up to 50%. Interviewees expect that a rise in applications will be seen at a later stage, because people often wait before applying for food assistance, which is also mentioned by a volunteer of a local food bank:

*“I also generally think that effect will be noticed much later. If people lose their jobs, they apply for social benefits. And it takes weeks or months before you think ‘hey, I have a structural shortage of money. I'll just go to the food bank.’ I do expect that increase [in recipients], but I think that is really an issue that will only come months later.”* (Local food bank 2)

Substantial shifts in actors were identified by the interviewees, indicating that the system of food aid showed adaptive capacity. Such shifts were enabled by the other sources of support for the work of food banks. While reliance on volunteers has been suggested to make food banks vulnerable in times of crisis, in fact new, and younger volunteers with plenty of free time quickly filled the gaps left by older volunteers who had to stay home. In addition, both national and local governments stepped in, as well as a host of sponsors to ensure the continuation of emergency food provided by food banks.

### Resources—shifts from surplus food to monetary donations

Food donations are the main resource for food banks. The influx of food has shown to be volatile. Donations of surplus food decreased at the start of the COVID-19 outbreak, because people were stockpiling and less food was left in supermarkets for food banks. Additionally, food donations decreased and fundraiser events at i.e. churches and schools were cancelled. While this source of food was squeezed at the start of the pandemic, interviewees also noticed an increase in new sources of food within the first months of the pandemic. This shift in food supply is for example explained in the following quote:

*“But fairly shortly afterwards [hoarding] there was a gigantic and somewhat unstructured supply because the catering industry closed. That happened on a weekend. So, Monday morning there were all kinds of supplies from closed catering establishments and nurseries and so on, such as fruit, dairy, vegetables and so on. Then we had a short period of two, three weeks that we received a huge amount of food for that reason. In the meantime, it has all returned to normal.”* (Local food bank 2)

While the amount of food donated to food banks was quickly restored through new sources of food, the kinds of donations differed and not all food was suitable for distribution through food banks, because they do not have the facilities to repackage the large quantities and partly processed food.

Next to new sources of food, the COVID-19 pandemic resulted in a significant increase in monetary donations, partly due to their own publicity efforts. A volunteer from a regional distribution centre explained the increased monetary donations and the increased costs during the COVID-19 pandemic:

*“There has been a lot of publicity and it continues to this day. As a result, we receive more [monetary] donations...There are foundations that come to us with the question if they can help us, that some municipalities say that we can use a part of an emergency fund. That is very nice, because it is all hands-on now and the costs are increasing. You need of course all the plastic packaging, we had to extend our conveyor belt a bit to be able to stand far apart.”* (Distribution centre 1)

Food banks adapted to the new donations. They spent more time on repackaging food and used the influx of monetary donations to realize such adaptations, such as in allowing the purchase of packaging material. Some food banks distributed monetary donations to their recipients in the form of vouchers they could use to purchase food in supermarkets. However, it is unclear whether monetary donations will be a reliable resource after the first period of the COVID-19 pandemic.

### Processes—pragmatism and innovation

Food distribution practices were adapted to reduce the contact moments and minimise the risk of spreading COVID-19. Food banks enforced a 1.5 m distance between individuals, used screens where the 1.5 m was difficult, used hand disinfectant regularly, and allowed a limited number of people at the food bank. Sometimes such adaptations were not feasible and other solutions were sought. As discussed above, some food banks offered food vouchers instead of food. On the one hand, food banks switched to supermarket vouchers to minimise the contact moments and thus reduce the risk to spread COVID-19. On the other hand, the supermarket vouchers were an alternative form of food assistance when there was insufficient food available. The vouchers were often donated by a supermarket, and recipients could then use the voucher at the specific supermarket. Multiple food banks also decided to deliver the food parcels to the recipients' homes to minimise the number of people at the food banks. Other food banks switched to issuing food in certain time blocks, while extending the total time for distribution. A volunteer from a local food bank mentioned that the extend time for distribution was perceived as very positive:

“*Well, what might be nice is that at first people thought that how we had organized it was fine. But by working with times now, it is a lot quieter, and it is not as hectic and it is easy to oversee. And the volunteers also think ‘hey, this is also quite relaxed’. That is experienced as very positive and that is why I wonder whether that will be reversed, ever. But as things are going now and how it is organized, this is experienced as very positive.”* (Local food bank 7)

Other interviewees also mentioned the benefit for recipients due to the reduced waiting time for picking up food parcels. Therefore, the use of set time blocks may be continued after COVID-19.

COVID-19 also caused changes in the intake procedure, such as conducting them online or even halting them for the time being. Multiple food banks eliminated intakes and re-intakes to use that time for other processes to continue food assistance. This means that people who applied for food assistance were accepted immediately before assessing their eligibility for food assistance. While local food banks are responsible for admission, the change was advised by the national organization.

*“And back in March, we immediately said: just stop the re-intake, postpone it. That takes energy and time. And you are unable to meet the people in person, so just let them continue [the food assistance]. You might have more clients [as recipients are called by Dutch food banks], but oh well. Also concerning the intake: let’s help those people who apply for food assistance and later on assess if they are eligible for food aid. At most they receive something for a couple of weeks which they are not entitled to. But okay, it is what it is. We are not the government who has to justify every penny.”* (Voedselbanken Nederland)

In sum, local food banks showed organizational resilience by adapting their procedures for distribution and intake, allowing them to ensure a continuation of food aid. Such changes are sometimes temporary but may lead to more long-term changes. An important factor is the fact that the organization is not a government agency and does not have to account for how they distribute the food, nor for whom they serve. Decisions can be made locally, though the national organization seems to have offered suggestions on how to approach challenges.

### Functions – prioritizing food provisioning

While providing food to households in need is a core function of all food banks, reducing food waste and offering a place for social interaction were seen as important functions as well before the corona pandemic (van der Horst et al., 2020). During the first months of the pandemic, a main emphasis was put on food provisioning over the other functions in the adaptations that were made, such as the issuing of gift cards.

*“But because we had an empty storage and we had the question of how we could reduce those contact moments, we were a bit confused. And many food banks closed. And we were like we are in [large city] and we are too big [to fail]. Too many people depend on us, and we cannot afford to close. So, then we had a few weeks, I think four weeks, we issued supermarket gift cards.”* (Local food bank 4).

Interviewees noted that the emphasis on facilitating social interaction decreased dramatically. To minimise the risk of spreading COVID-19, food banks chose to limit the number of people at food banks or not allow people inside. Consequently, the food banks were functioning less as a social space where people meet and tell their story.

*“... the visit to the food bank also has a very important social function for many people. And that was especially noticeable during the corona crisis in which people had to keep their distance and the contact moments were very much minimised. That is what I heard from distribution points. I was told that people thought that was a great pity, because for many people financial poverty goes hand in hand with social poverty.”* (Local food bank 4)

While food banks aim to facilitate various functions—including reducing the social isolation of people in poverty and the reduction of food waste—during the first months of the pandemic food aid was prioritized. Through adapting the functions and reducing the importance of the other functions, the main aim of providing food aid was best safeguarded. From the perspective of the contribution of food banks to a system of food aid, this contributed to resilience.

## Conclusion

Based on interviews with food bank volunteers across local, regional and national levels in the Netherlands we aimed to understand the resilience and further entrenchment of food aid through food banks. We unravelled the ability of the particular food aid system to absorb shocks, adapt to new circumstances or transform into a new system. While there were certainly some problems in the continuation of services, the system of food assistance proved largely resilient through its adaptive capacity towards variable donations, drop and influx of volunteers, COVID-19 health risks and measurements, and increased demand. This capacity was found in part in the organization of food banks. Ways of working were adapted at local food banks, e.g. handing out supermarket vouchers and organizing food distribution in time slots, while maintaining their role in providing food aid. Such adaptive capacities were supported by the other levels, for example through a national organization advising local food banks to pause the re-intake process. But continuity of food aid was also achieved through food banks being embedded in a broader system of food aid where other actors stepped in to offer support. The latter can be ascribed to the food banks’ publicity efforts and collaboration with external partners, while maintaining an organizational structure and autonomy that allows local food banks to make ad hoc decisions.

Because of the dominance of food banks as the principal form of food assistance in the Netherlands, it was deemed “too big to fail” as an interviewee stated, not only by food bank volunteers, but also by actors who stepped in to offer support to ensure continuation of services. New donors offered money and thereby replaced some food donations that had become less reliable. Younger people applied as volunteers and replaced the older volunteers who were forced to shield. Local, as well as national governments, also offered to help in case the food banks did not manage to continue food aid without their support. Though the importance was downplayed in an interview, the deployment of military personnel to help in distribution centres was testament to this status as being perceived as “too big to fail”. That the size of a food bank system organized at national level contributes to their resilience has also been argued by Baker and Russell (2020) in accordance to the Trussel Trust food banks in Britain. They state that the large organisational capacity of Trussel Trust allowed development of existing partnerships and new ones, which helped them to continue their operations during the corona pandemic.

The challenges Dutch food banks faced and the adaptive capacity of the food aid system of which Dutch food banks are part of, as noticed in this research, show similarities with a case study by Penco et al. (2021) about the Italian organization of food banks (the FBAO). Their research emphasizes the innovative practices adopted by Italian food banks during the first months of the pandemic through experimentation with procedures (e.g. recruitment of younger volunteers, handing out gift cards) and collaboration with external partners, which enabled them to quickly respond to challenges such as changing food supplies, safety of volunteers and recipients, and increased demands. While every crisis brings its own specific challenges, the adaptive capacity of the particular food aid system we explored in the context of the corona pandemic also aligns with insights of the research by Coque and González-Torre (2017) about food banks in Spain in relation to the economic crisis that started in 2007–2008. Both studies point to the importance of connections with external organizations and the widespread appreciation for food banks for the adaptive capacity of food banks in a context of a crisis.

Although Dombroski et al. (2020) indicate that the COVID-19 context provides chances to re-think existing food practices, including responses to food insecurity, this research demonstrates how the corona pandemic entrenches food banks as central to the Dutch food aid system. Such broad support for people in need funnelled through food banks could be seen as an indication of social solidarity, which Elcheroth and Drury (2020) deem vital to further resilience of a society beyond the crisis. While support for food banks is indeed an expression of social solidarity, it is also an indication of the dominance of food aid through food banks as central to the understanding and practices of ensuring food security. This approach remains vulnerable. Interviewees express worries that the number of people applying for food aid will rise in the coming months and years, when household financial buffers are depleted and social security measures that have been implemented during the pandemic are phased out. At the same time, interviewees also indicate that the donation of sufficient food remains unreliable. While the system of food aid appeared resilient during the first shock of the pandemic, a truly resilient societal response would be to simultaneously mend the problems that lead to growing inequality, poverty and food insecurity. In other words, while the embeddedness in a broad system and the level of support contributed to the continuation of food aid, further reliance on and entrenchment of food banks should not go unchallenged.

## Data Availability

Not applicable.
